# APOE genotype‐dependent effects of enhanced BMP signaling on astrocytes and co‐cultured neurons

**DOI:** 10.1002/alz.090956

**Published:** 2025-01-03

**Authors:** Anne K Linden, Amira Affaneh, John A Kessler, Chian‐ Yu Peng

**Affiliations:** ^1^ Northwestern University, Chicago, IL USA

## Abstract

**Background:**

Apolipoprotein E4 (E4) is the strongest genetic risk factor for sporadic Alzheimer’s Disease (AD), and aging is the greatest overall risk factor for AD. Many cellular and molecular changes occur within the brain throughout aging, one of which being the increased bone morphogenetic protein 4 (BMP4) signaling. As APOE and BMPs are known to interact in non‐neuronal organs, we hypothesized that enhanced BMP signaling in the brain may interact with APOE in a genotype‐dependent manner to initiate or exacerbate neuropathological cascades relevant to AD.

**Method:**

E4 patient‐derived IPSC, along with CRISPR‐corrected isogenic APOE3 (E3) controls, were virally induced into astrocytes with SOX9 and NFIB overexpression, or neurons with NGN2 overexpression, using previously published protocols. After maturation, astrocytes and neurons cultured alone or in co‐culture, were treated for 72 hours BMP4 or vehicle prior to collection to observe changes.

**Result:**

In the presence of BMP4, E4 astrocytes accumulated larger amounts of lipids in the cytoplasm compared to E3 astrocytes. This accumulation of lipids resulted from enhanced lipid generation, reduced lipid breakdown, and reduced efficiency of beta oxidation compared to E3 astrocytes treated with BMP4. Further, single cell transcriptomic analysis of astrocyte‐neuron co‐cultures revealed that BMP4 promotes astrocyte to neuron cholesterol signaling from E4 astrocytes more than E3 astrocytes, and BMP4 treatment reduces elevated TGFβ signaling from E4 astrocytes to co‐cultured neurons. These BMP4 induced changes coincide with enhanced phosphorylation of tau and reduced oxidative phosphorylation capacity in neurons cocultured with E4 astrocytes compared to E3 astrocytes.

**Conclusion:**

Increased BMP signaling alters the physiology of astrocytes in an APOE‐dependent manner, with E4 astrocytes exhibiting more abnormalities in lipid metabolism than E3 astrocytes. Specifically, BMP‐treated E4 astrocytes have greater lipid droplet accumulation due to increased lipid generation, reduced lipid breakdown, and lack of beta oxidation when compared to E3 astrocytes treated with BMP4. E4 astrocytes exert greater effects than E3 astrocytes on the disease phenotype of co‐cultured AD neurons, with increases in phosphorylated tau and decreases in oxidative phosphorylation efficiency. This suggests that enhanced BMP4 signaling in the aging brain may be one of the contributing factors to development of AD.

